# Dietary Protein-Induced Changes in Archaeal Compositional Dynamics, Methanogenic Pathways, and Antimicrobial Resistance Profiles in Lactating Sheep

**DOI:** 10.3390/microorganisms13071560

**Published:** 2025-07-02

**Authors:** Maida Mushtaq, Xiaojun Ni, Muhammad Khan, Xiaoqi Zhao, Hongyuan Yang, Baiji Danzeng, Sikandar Ali, Muhammad Hammad Zafar, Guobo Quan

**Affiliations:** 1 Yunnan Animal Science and Veterinary Institute, Jindian, Panlong District, Kunming 650000, China; maidach.17@gmail.com (M.M.); xiao666jun@163.com (X.N.); khanbwn011@gmail.com (M.K.); zhao20220311@163.com (X.Z.); yang20211101@163.com (H.Y.); baiji20190101@163.com (B.D.); 2 Yunnan International Joint Laboratory of Conservation and Innovative Utilization of Sheep Germplasm Resources, Jindian, Panlong District, Kunming 650000, China; sikandar.ptbav@gmail.com; 3 Yunnan Provincial Engineering Research Center of Animal Genetic Resource Conservation and Germplasm Enhancement, Panlong District, Kunming 650000, China; 4 Yunnan Provincial Genebank of Livestock and Poultry Genetic Resources, Panlong District, Kunming 650000, China; 5 Dow Institute for Advanced Biological and Animal Research, Dow University of Health Sciences, Ojha Campus, Karachi 74200, Pakistan; 6 College of Animal Science and Technology, Yangzhou University, Yangzhou 225012, China; hammadzafar075@gmail.com

**Keywords:** sheep, gut archaea, dietary protein, methane emission, antimicrobial resistance, metagenomics

## Abstract

Dietary protein levels greatly influence gut microbial ecosystems; however, their effects on gut archaea and associated functions in ruminants require further elucidation. This study evaluated the impact of varying dietary protein levels on gut archaeal composition, antimicrobial resistance (AMR) genes, virulence factors, and functional capacities in sheep. Eighteen ewes (Yunnan semi-fine wool breed, uniparous, 2 years old, and averaging 50 ± 2 kg body weight) were randomly assigned to diets containing an 8.5 (low; H_1), 10.3 (medium; H_m), or 13.9% (high; H_h) crude protein level from the 35th day of pregnancy to the 90th day postpartum. The total duration of the experiment was approximately 202 days. A total of nine fecal samples (three from each group) were analyzed via 16S rRNA and metagenomics sequencing. Higher archaeal alpha diversity and richness were observed in the H_m and H_h groups compared to the H_l group (*p* < 0.05). A Beta diversity analysis revealed the archaeal community’s distinct clustering mode based on protein levels. The methanogenic genera *Methanobrevibacter* and *Methanocorpusculum* were dominant across the three groups, and their abundance was influenced by protein intake. A functional prediction analysis indicated moderate changes in amino acid and carbohydrate metabolism, which are particularly associated with methane production, an important source of greenhouse gases. AMR genes (e.g., *tetA* (60), *patA*, *vat*, and *Erm methyltransferase*) and virulence factors (*Bacillibactin*, *LPS*) were significantly enriched when animals were fed high-protein diets. Our results demonstrated that dietary protein levels significantly influence gut archaeal composition, AMR gene enrichment, and related functional pathways. Medium-protein diets promoted greater archaeal diversity, whereas high-protein diets favored resistance gene proliferation and enhanced methanogenic activity. Optimizing dietary protein intake may enhance gut health, mitigate antimicrobial resistance risk, and reduce methane emissions, thereby supporting livestock sustainability and environmental protection.

## 1. Introduction

In ruminants, the rumen microbiota play crucial roles in digesting and fermenting feedstuffs, thereby influencing animal health, productivity, and environmental emissions [1]. The archaeal population is mainly involved in methane production through anaerobic digestion processes [2]. It is well known that dietary composition, particularly protein content, is one of the major factors shaping the structure and function of rumen and intestinal microbial communities in ruminant species [3,4], supplying peptides, amino acids, and ammonia [5]. Thus far, alterations in protein levels can modulate microbial diversity and metabolic functions, potentially affecting fermentation end-products, nitrogen utilization efficiency, and greenhouse gas emissions [6]. Moreover, protein is the costliest segment of ruminant nutrition, and higher dietary protein content leads to nitrogenous waste and environmental contamination [7,8]. Despite the growing body of research on bacteria and protozoa, relatively less attention has been given to archaea [9], particularly concerning dietary protein variations during early lactation, a physiologically demanding period in ewes.

Archaea in ruminants are traditionally considered detrimental due to methane emissions and associated energy losses [10]. However, their hydrogen-consuming capacity may offer benefits, as hydrogen accumulation inhibits rumen and gut fermentation [10]. Li and Guan [11] reported significant *Methanomassiliicoccales* enrichment in the rumen of beef cattle with superior feed efficiency and later established positive associations between several archaeal species, including *Methanobrevibacter smithii*, and feed utilization [12]. Similarly, Li et al. [13] and McLoughlin et al. [14] identified three *Methanobrevibacter species* linked to enhanced feed efficiency in cattle and sheep, respectively. Another study by Shen et al. [15] demonstrated that goat gut archaea contribute to hydrogen reduction and carbohydrate metabolism through carbohydrate-active enzyme (CAZyme) gene expression. Studies in cattle and goats have shown that dietary protein levels can influence ruminal archaea, with higher protein sometimes linked to shifts in methanogen diversity and methane production [4,16,17,18]. Similarly, factors like forage-to-concentrate ratio have been reported to significantly alter archaeal populations in sheep and cattle [19,20]. While these findings highlight the important yet underexplored role of archaea in livestock digestion, studies specifically evaluating the impact of dietary protein on fecal archaeal communities remain limited.

As a non-invasive tool, fecal microbiota profiling offers valuable insights into the hindgut and, indirectly, ruminal archaea [21]. Understanding how fecal archaeal communities respond to dietary protein changes is critical for improving nutrient utilization, boosting productivity, and mitigating methane emissions, an environmental concern in ruminant production [22,23]. Advances in meta-omics, such as high-throughput technologies like 16S rRNA sequencing and metagenomics [24], now allow the detailed characterization of archaeal diversity and functions. With all of this in mind, the current study hypothesizes that dietary protein level may affect fecal archaeal communities, functional pathways, or gene expression in early lactating Yunnan semi-fine wool ewes. In this context, the present study aimed to investigate the effects of three different dietary protein levels on fecal archaeal richness, taxonomic composition, functional pathways, and differential gene expression in early lactating ewes. These insights could guide nutritional strategies to enhance productivity and promote environmental sustainability.

## 2. Materials and Methods

### 2.1. Ethics Approval, Experimental Site, Research Design, Husbandry Practices, and Fecal Sample Collection

All procedures were approved by the Yunnan Animal Science and Veterinary Institute Ethical Committee (201911004) and followed the guidelines of the State Science and Technology Commission of China (1988), as well as the Yunnan Provincial People’s Congress (2007.10), with strict adherence to approved protocols. The feeding experiment to estimate the influence of dietary protein levels on fecal archaeal composition in lactating ewes was conducted at the Yunnan Animal Science and Veterinary Institute farm, Kunming, China (26°22′ N; 103°40′ E). For this, eighteen ewes (Yunnan semi-fine wool breed, uniparous, 50 ± 2 kg body weight, 2 years old) were randomly allocated to three groups receiving diets containing an 8.5 (low; H_1), 10.3 (medium; H_m), or 13.9% (high; H_h) protein level, following a completely randomized design. The protein levels were determined by considering the nutritional requirements of the lactating sheep as set out by the Yunnan Sheep Production Commission, China. The chemical compositions of the dietary ingredients (procured from the local market) and treatments were determined by following the established protocols of the International [25]. The detailed formulation and chemical composition are given in Table 1. During the experimental period, ewes were housed individually (pens equipped with separate feeders and water supply) in a shed with identical farming conditions. The ewes were fed twice daily (at 08:00 and 14:00 h during the day) to ensure the ad libitum intake of their respective diets from the 35th day of pregnancy until the 90th day postpartum (approximately 202 days), with free access to fresh water. On the 90th day, three sheep from each group (according to feed intake) were randomly subjected to fecal sample collection from the terminal rectum, using sterile techniques as described by Zhao et al. [26]. Approximately 10 g of feces was placed in sterile 10 mL freezing tubes (BIOFIL, Guangzhou, China) and immediately frozen in liquid nitrogen before being stored at −80 °C for further analysis.

### 2.2. DNA Extraction

On the 5th day of collection, total DNA was extracted from fecal samples using the E.Z.N.A.^®^ Stool DNA Kit (D4015, Omega, Bio-tek, Inc., Norcross, GA, USA), following the manufacturer’s instructions. Ultrapure water was used as the blank control. The DNA was eluted in 50 μL of elution buffer and stored at −80 °C for subsequent analysis.

### 2.3. Polymerase Chain Reaction, Amplification, and 16S rDNA Sequencing

The V3-V4 region of the prokaryotic 16S rRNA gene was amplified using the primers 341F (5′-CCTACGGGNGGCWGCAG-3′) and 805R (5′-GACTACHVGGGTATCTAATCC-3′). Each sample was tagged with unique barcodes for identification. The polymerase chain reaction (PCR) mixture (25 μL) included 25 ng of template DNA, 12.5 μL of PCR Premix, 2.5 μL of each primer, and PCR-grade water to achieve the final volume. PCR conditions included an initial denaturation at 98 °C for 30 s, followed by 32 cycles of denaturation at 98 °C for 10 s, annealing at 54 °C for 30 s, extension at 72 °C for 45 s, and a final extension at 72 °C for 10 min. The PCR products were analyzed using 2% agarose gel electrophoresis. Negative controls with ultrapure water were included to exclude false positives. The purified PCR products were quantified using a Qubit fluorometer (Invitrogen, Waltham, MA, USA). Amplicon libraries were prepared and sequenced using the Illumina NovaSeq PE250 platform.

### 2.4. Metagenomics Sequencing

The three samples from each treatment group underwent metagenomic sequencing, yielding nine samples for further sequencing. DNA libraries were constructed using the TruSeq Nano DNA LT Library Preparation Kit (No: FC-121-4001, Illumina, Inc., San Diego, CA, USA). DNA fragmentation was performed using dsDNA fragmentase (No: NEB, M0348S, New England Biolabs Japan Inc., Tokyo, Japan) for 30 min at 37 °C. After fragmentation, blunt-end DNA was generated, and adapters were ligated to the DNA fragments. Amplification was performed with the following PCR conditions: initial denaturation at 95 °C for 3 min, followed by 8 cycles of denaturation at 98 °C for 15 s, annealing at 60 °C for 15 s, and extension at 72 °C for 30 s, with a final extension at 72 °C for 5 min. Paired-end 2 × 150 bp sequencing was performed on the Illumina HiSeq 4000 platform according to the manufacturer’s instructions.

### 2.5. Bioinformatics Analysis

Raw sequencing reads were processed by removing sequencing adapters using Cutadapt v1.957. Low-quality reads were trimmed using fqtrim v0.94, and host contamination was removed by aligning the reads to the host genome using Bowtie2. The quality-filtered reads were assembled de novo using IDBA-UD to construct metagenomes for each sample. Coding regions were predicted using MetaGeneMark v3.26, and the resulting unigenes were clustered using CD-HIT v4.6.160. Unigene abundance was estimated by Transcripts Per Million using Bowtie2. Taxonomic assignment was performed using DIAMOND v0.7.1261, aligning with the NCBI NR database. Functional annotations of the unigenes were obtained using the comparative antibiotic resistant (CARD), virulence factor (VFDB), and Kyoto Encyclopedia of Genes and Genomes (KEGG) databases. A differential analysis of taxonomic, functional, or gene-level abundance was performed using Fisher’s exact (for non-replicated groups) or Kruskal–Wallis tests (for replicated groups).

## 3. Results

### 3.1. Sequencing Reads

The sequencing results indicate high-quality data across all groups (H_m, H_h, and H_l) (Table S1). Group H_m generated 126 million raw reads, with a valid ratio of 98.43%, a Q20 of 99.66%, and a Q30 of 98.18%; H_h produced 137 million raw reads, yielding a valid ratio of 98.28%, a Q20 of 99.68%, and a Q30 of 98.28%; and H_l resulted in 119 million raw reads, with a valid ratio of 98.23%, a Q20 of 99.70%, and a Q30 of 98.35%. The GC content was consistent across all groups, ranging from 44.50% to 44.67%. Overall, all groups exhibited excellent sequencing performance, with high valid read ratios and quality scores, ensuring robust and reliable data.

### 3.2. Fecal Archaeal Richness

The archaeal alpha diversity indices revealed that dietary protein levels influenced the gut microbiota composition in sheep (Table 2). The Shannon diversity index was higher in the medium (4.79 ± 0.26) and high-protein (4.28 ± 0.67) groups compared to the low-protein (4.30 ± 0.59) group (*p* < 0.05). Similarly, species richness indices (observed species and Chao1) were significantly greater in the high- (389 ± 39; 426 ± 24) and medium-protein (395 ± 9; 418 ± 4) groups compared to the low-protein group (298 ± 26; 318 ± 21) (*p* < 0.05). Simpson’s indices (>0.88) and goods coverage (>0.999) across all groups indicated a high level of community evenness and sufficient sequencing depth.

**Figure 1:**
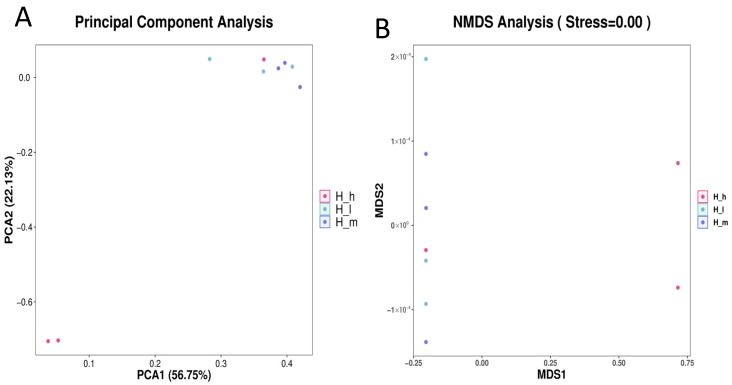
Principal coordinate (**A**) and NMDS (**B**) plots for beta diversity analysis of archaeal composition in fecal samples of lactating ewes fed low (H_l), medium (H_m), and high (H_h) dietary protein levels.

Beta diversity analyses showed a clear separation of archaeal communities, suggesting that variations in dietary protein levels significantly influenced the beta diversity of the archaeal community (Figure 1). Principal component (PC) analysis demonstrated that PC1 explained 56.75% of the total variance, highlighting the strong effect of protein level on the gut archaeal composition, while PC2 explained 22.13% of the variance (Figure 1A). A non-metric multidimensional scaling analysis (stress = 0.00) further confirmed distinct clustering among the groups, with a greater shift observed under the high-protein diet (Figure 1B).

### 3.3. Archaeal Taxonomic Profiling

**Figure 2:**
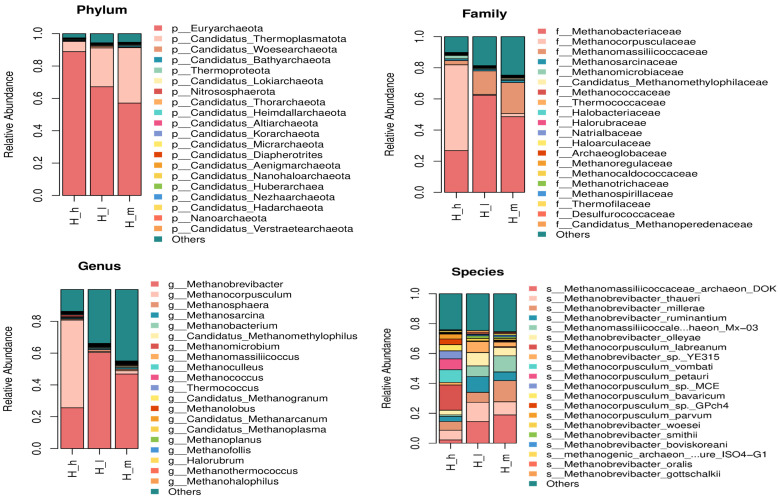
Fecal archaeal toxemic relative abundance profiling (stacked bar charts: phylum; family; genus; and species, respectively) of early lactating ewes fed low (H_l), medium (H_m), and high (H_h) dietary protein levels.

The fecal archaeal community composition differed significantly among the three dietary protein groups (H_m, H_h, and H_l), at both the phylum and genus levels (Figure 2). At the phylum level, *Euryarchaeota* was the dominant phylum across all groups; its relative abundance was highest in the H_m group—accounting for over 85% of the archaeal sequences—compared to the H_l and H_h groups, where the abundance slightly decreased, accompanied by an increased representation of minor phyla such as *Thermoplasmatota* and *Crenarchaeota*. Notably, the H_h group exhibited the greatest phylum-level diversity. At the genus level, *Methanobrevibacter* was the predominant genus in all dietary treatments (*p* < 0.05). Sheep receiving the medium-protein diet had a significantly higher proportion of *Methanobrevibacter* (approximately 75–80%) compared to the low- and high-protein groups. At the species level, *Methanocorpusculum labreanum*, *Methanocorpusculum unclassified*, and *Methanobrevibacter smithii* were consistently dominant across all dietary groups. High-protein diets resulted in significantly higher relative abundances of *M. labreanum* and *M. smithii* compared to the low- and medium-protein groups (*p* < 0.05). In contrast, medium-protein diets promoted the enrichment of minor archaeal species, including *Methanogenic archaeon ISO4-G1*, *Candidatus Bathyarchaeota archaeon MX-06*, and *Methanomassiliicoccus luminyensis* (*p* < 0.05). These findings indicate that moderate dietary protein intake supports a more diverse and balanced archaeal community structure in the sheep gut.

### 3.4. Comparative Taxonomic Profiling of Fecal Archaeal Species

**Figure 3:**
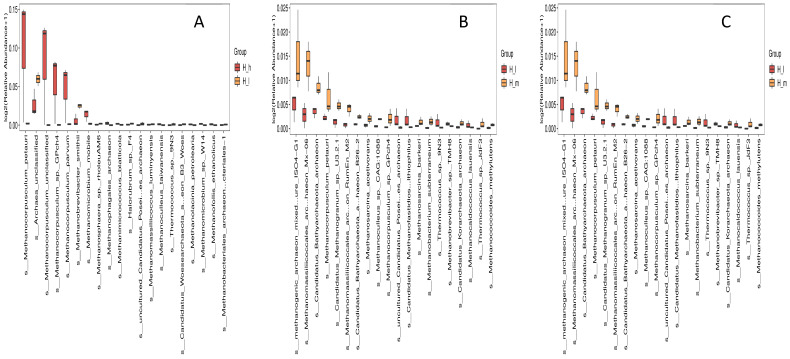
Species-level pairwise comparisons of fecal archaeal toxemic relative abundances in early lactating ewes receiving low (H_l), medium (H_m), and high (H_h) dietary protein levels, presented as box plots ((**A**): H_h vs. H_l; (**B**): H_h vs. H_m; (**C**): H_l vs. H_m).

*Methanocorpusculum labreanum*, *Methanocorpusculum* unclassified, and *Methanobrevibacter smithii* dominated the archaeal species across all dietary groups (Figure 3). Comparing the H_h and H_I groups (Figure 3A), *Methanocorpusculum labreanum* and *Methanobrevibacter smithii* exhibited significantly higher relative abundances in the former compared to the latter (*p* < 0.05). In contrast, a comparison between the H_I and H_m groups (Figure 3B) showed a significant increase (*p* < 0.05) in the relative abundances of Methanogenic archaeon mixed_urea ISO4-G1, *Candidatus Bathyarchaeota* archaeon MX-06, and *Methanomassiliicoccus luminyensis* in the latter. Additionally, a broader range of minor archaeal species were detected at higher levels in the H_m group compared to the H_I group (Figure 3C). Species richness and evenness also appeared to be greater in the former, suggesting a more diverse archaeal community structure under moderate protein intake.

### 3.5. Functional Prediction of Archaeal Communities

**Figure 4:**
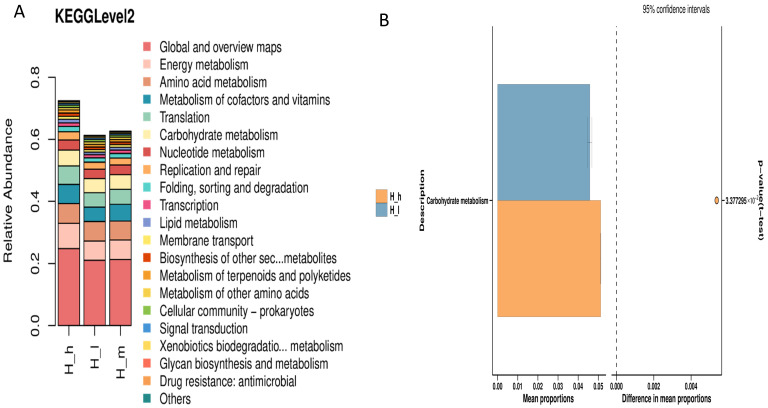
KEGG level 2 analysis (**A**) and comparison of abundance of genes involved in KEGG pathways ((**B**): H_h and H_l groups) for early lactating ewes receiving low (H_l), medium (H_m), and high (H_h) dietary protein levels.

The KEGG pathway analyses revealed functional differences in the fecal archaeal communities of sheep fed varying protein levels. The KEGG level 2 indicated subtle but notable variations, with mean proportion differences ranging from 0.00 to 0.05, suggesting that high-protein diets moderately altered pathways associated with amino acid and carbohydrate metabolism (Figure 4). These findings collectively suggest that dietary protein levels influence archaeal composition, as evidenced by principal component/non-metric multidimensional scaling analysis. They also suggest their functional potential, although the effect sizes remained modest.

### 3.6. Resistome Analysis

**Figure 5:**
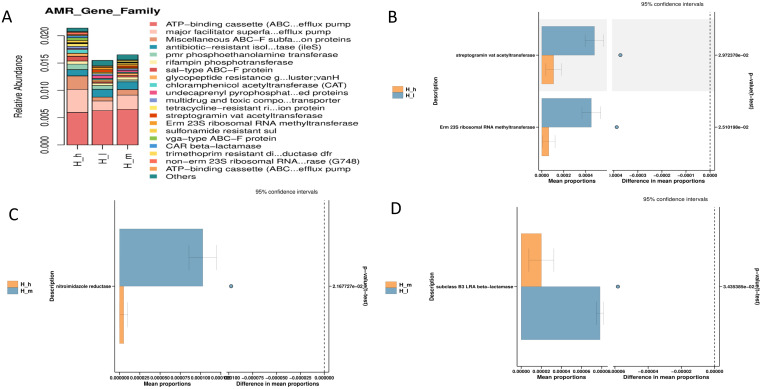
Antimicrobial resistance (AMR) gene profiles within sheep gut microbiota (**A**) in fecal samples from different groups of early lactating ewes receiving low (H_l), medium (H_m), and high (H_h) dietary protein levels, as well as a comparative analysis ((**B**): H_h vs. H_l; (**C**): H_h vs. H_m; (**D**): H_l vs. H_m).

**Figure 6:**
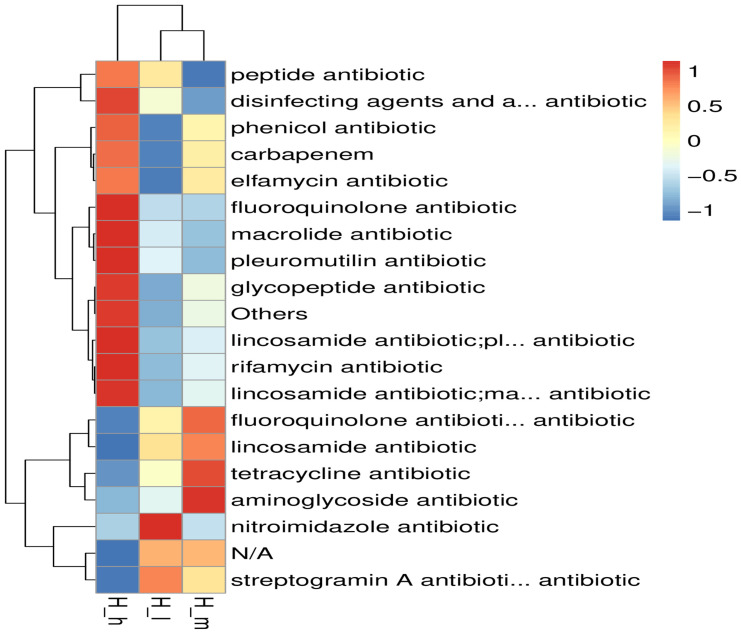
Comparative antibiotic-resistant database (CARD) analysis of fecal samples from different groups of early lactating ewes receiving low (H_l), medium (H_m), and high (H_h) dietary protein levels.

Dietary protein levels significantly influenced the antimicrobial resistance gene profiles within the sheep gut microbiota, as revealed by transcriptomic analyses and CARD comparisons (Figure 5 and Figure 6, respectively). A comparative analysis showed a considerable number of differentially expressed unigenes across groups (H_h vs. H_l: 1646 upregulated, 841 downregulated; H_h vs. H_m: 1258 upregulated, 209 downregulated; H_m vs. H_l: 1646 upregulated, 133 downregulated; *q* < 0.05). A BacMet database analysis indicated an enrichment of efflux pump systems (log2FC = 2.1, *q* = 0.02) and tetracycline resistance mechanisms (log2FC = 1.8, *q* = 0.03) in high-protein diets (Figure 6). Stress response genes were notably associated with low-protein diets (log2FC = −1.5, *q* = 0.04). Furthermore, a specific ARO analysis identified the tetracycline resistance gene tetA (60) (4.20 × 10^2^) and multidrug efflux pump gene patA (3.91 × 10^2^) as the most differentially abundant between high- and low-protein groups. High-versus medium-protein comparisons highlighted the enrichment of alternative tetracycline resistance genes (tetA (58): 4.74 × 10^2^) and efflux pumps (etrA: 3.78 × 10^2^), while medium-versus low-protein diets revealed shifts in β-lactam resistance profiles (LRA-8: 3.44 × 10^2^). A CARD database analysis corroborated these findings, showing a significant enrichment of vat acetyltransferase (2.97 × 10^2^) and Erm 23S ribosomal RNA methyltransferase (2.51 × 10^2^) under high-protein conditions. Hierarchical clustering further confirmed distinct AMR gene grouping patterns by dietary protein level. These findings suggest the protein-dose-dependent selection of AMR mechanisms, with high-protein diets favoring clinically relevant resistance to tetracyclines, macrolides, and β-lactams. This underscores the need for cautious dietary formulations in livestock systems to mitigate resistome gene propagation.

### 3.7. Virulence Factor Profiles

**Figure 7:**
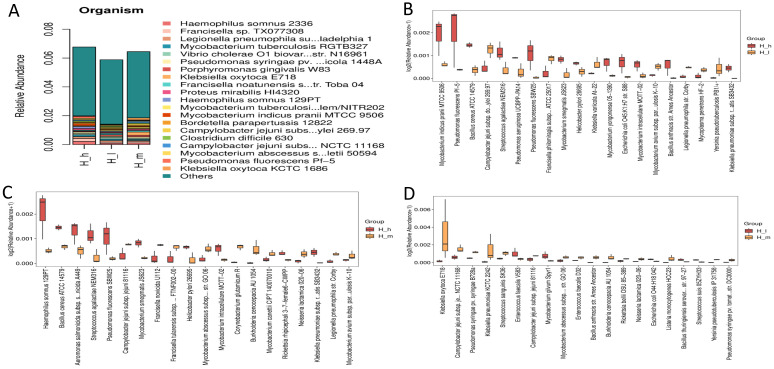
Virulence factor database analysis (VFDA) for organism abundance (**A**) in fecal samples from different groups of early lactating ewes receiving low (H_l), medium (H_m), and high (H_h) dietary protein levels, as well as a comparative enrichment analysis ((**B**): H_h vs. H_l; (**C**): H_h vs. H_m; (**D**): H_l vs. H_m).

An analysis of the virulence factor profiles using the VFDB database demonstrated that dietary protein levels significantly influenced the virulence potential of fecal archaea (Figure 7). Relative to low-protein diets (H_l), high-protein diets (H_h) led to a marked upregulation of iron acquisition systems, such as Bacillibactin (Δ = 4.20 × 10^2^), and immune-modulating factors, such as LPS (Δ = 1.35 × 10^2^), suggesting enhanced microbial competition and altered host–microbe interactions. Medium-protein diets (H_m) showed intermediate patterns, with prominent contributions from Enterobactin (Δ = 4.68 × 10^−2^) and phytotoxin-related genes. The attenuation of VF disparities across H_h, H_m, and H_l groups underscores dietary protein as a key driver of archaeal virulence strategies, particularly in iron-limited gut environments. These findings underscore the significance of dietary protein in shaping the functional ecology of archaea, highlighting the need for further metatranscriptomic validation.

### 3.8. Differential Expression of Gut Microbial Functional Pathways

**Figure 8:**
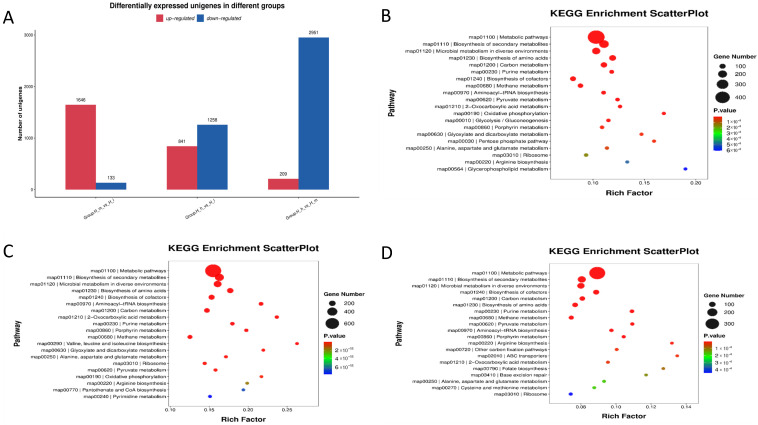
Differential expression of unigenes (bar graph (**A**)) in fecal samples from different groups of early lactating ewes receiving low (H_l), medium (H_m), and high (H_h) dietary protein levels, as well as a comparative KEGG enrichment scatter plot analysis ((**B**): H_h vs. H_l; (**C**): H_h vs. H_m; (**D**): H_l vs. H_m).

Transcriptomic profiling further revealed that dietary protein levels significantly influenced gut microbial functional pathway expression (Figure 8). High-protein diets (H_h) were associated with a substantial upregulation of the genes involved in nutrient transport, energy metabolism, and amino acid biosynthesis pathways, whereas low-protein diets (H_l) exhibited upregulated stress responses, defense mechanisms, and carbohydrate metabolism pathways. The differential expression patterns observed across the three dietary groups (H_h, H_m, and H_l) suggest dynamic microbial functional adaptations in response to dietary protein availability, which may have significant implications for host health and feed utilization efficiency.

## 4. Discussion

The findings of this study underscore the significant impact of dietary protein levels on the fecal archaeal populations of early lactating ewes, revealing that protein intake directly influences intestinal microbial diversity, community composition, and functional potential. We observed that a higher dietary protein level was associated with increased archaeal diversity compared to moderate- and low-protein diets. This increase in archaeal diversity is likely due to the enhanced availability of nitrogen sources, such as amino acids and peptides, which are essential for microbial growth [6] and fermentation in the rumen [27]. These results are consistent with previous studies that have shown that increasing dietary protein can expand microbial diversity in small ruminants [28,29], thereby improving fermentation efficiency and nutrient utilization. The greater archaeal diversity observed in the high-protein group suggests a more robust microbial ecosystem capable of utilizing a broader array of substrates, which is critical for optimizing ruminal function and promoting sheep health [30].

As indicated by the beta diversity analysis, the shifts in microbial community composition also support the notion that dietary protein levels play a key role in shaping gut microbial populations. In this experiment, the archaeal populations revealed a significant increase in methanogens in the high-protein group. High-protein diets led to distinct archaeal profiles, specifically methanogenic archaea enrichment, and particularly the *Methanobrevibacter* genus. This finding aligns with previous studies that have demonstrated the positive relationship between dietary protein and methanogen abundance in the rumen [31,32]. The availability of fermentable energy substrates from a high-protein diet likely stimulates the growth of methane-producing archaea, as these microbes utilize the hydrogen produced during fermentation to generate methane [32]. Although methanogenesis is a natural part of rumen fermentation, it results in significant energy loss for the host animal, since the energy contained in methane is unavailable for metabolic use. Additionally, methane production is energetically costly and contributes to greenhouse gas emissions. In this study, *Methanosphaera* and *Thermoplasmata* genus enrichment was also observed in the high-protein group. These results are in line with previous studies [33,34] reporting that higher dietary protein contents encourage *Methanosphaera* and *Thermoplasmata* genus enrichment in ruminant species. Moreover, it is well established that these genera are involved in different aspects of ruminal metabolism [35]. These shifts suggest that dietary protein influences the functional capacity of the archaeal community, potentially altering the pathways involved in energy production, nitrogen fixation, and other critical metabolic processes. The upregulation of genes associated with methane production in the high-protein diet group supports this interpretation, as higher protein availability likely stimulates the expression of methane-related genes, which are central to the metabolic activity of methanogens [36]. While this upregulation of methane-producing pathways is a natural consequence of increased protein intake, it also highlights the need for future strategies aimed at mitigating methane emissions from ruminants, such as dietary modifications or the use of methane inhibitors [37]. Thus, while higher protein levels enhance microbial fermentation and animal productivity, they may also exacerbate methane emissions, posing challenges for both animal efficiency and environmental sustainability.

Functional prediction analyses further support the notion that dietary protein influences microbial metabolism in the gut, particularly in terms of amino acid metabolism, energy production, and methanogenesis [3,6]. In the high-protein diet group, we observed an enrichment of genes related to branched-chain amino acid metabolism, which are essential for microbial protein synthesis and are typically provided in greater quantities by high-protein diets [38]. This enrichment reflects the increased availability of amino acids for microbial growth, in turn promoting microbial protein synthesis and improved fermentation efficiency. However, the functional prediction data also indicated a concomitant increase in methane-related pathways, suggesting that while higher protein levels can enhance nutrient utilization in the rumen, they also drive methane production [39], thus presenting a trade-off between microbial efficiency and environmental impact.

A differential expression analysis revealed a large number of unigenes significantly regulated by dietary protein content, with the greatest contrasts observed between high- and low-protein groups. The enrichment of efflux pump systems and tetracycline resistance mechanisms under high-protein diets aligns with previous findings in mice [40], where high-protein or energy-dense diets have been linked to increased antibiotic resistance gene abundance. Notably, the upregulation of efflux pump genes such as patA and tetracycline resistance determinants like tetA suggests that a protein-rich gut environment may facilitate multidrug-resistant microbial population selection [40]. These findings are of particular concern, as efflux systems contribute to cross-resistance across multiple antibiotic classes, potentially complicating infection management strategies in animal production systems. The comparative enrichment of β-lactam resistance genes (e.g., LRA-8) between medium- and low-protein diets—and the higher abundance of macrolide resistance markers (*vat acetyltransferase*, *Erm 23S rRNA methyltransferase*) in high-protein diets—further indicates a broad-spectrum adaptation of the gut microbiota to protein availability [41]. Hierarchical clustering analyses confirmed the distinct grouping of AMR gene profiles by protein level, reinforcing the hypothesis that dietary interventions can significantly restructure the resistome landscape. These results underscore the importance of considering not just growth performance but also the microbiological and public health implications, when formulating high-protein rations for ruminants. Virulence factor profiling revealed an additional layer of microbial functional adaptation. High-protein diets significantly upregulated iron acquisition systems, such as *Bacillibactin*, and immune-modulating factors, such as lipid polysaccharides. These results are consistent with previous studies reporting that higher dietary protein levels upregulated this mechanism in pigs [42]. It is well known that iron acquisition is a critical virulence trait in microbial competition and host colonization [43]. Its enrichment suggests that high-protein diets may intensify microbial competition in the gut, possibly favoring pathogenic traits. The observed increase in LPS-related genes could potentially enhance gut inflammatory responses, though further validation through host inflammatory marker analysis is warranted. Given the emerging recognition of archaeal contributions to gut health and disease [44], our findings point to an underexplored area where dietary manipulation could inadvertently shape archaeal virulence potential. Collectively, these findings highlight dietary protein as a potent modulator of both AMR and virulence profiles in the gut microbiota of ruminants. From a livestock management perspective, the results advocate for the cautious balancing of dietary protein inputs, aiming to optimize animal performance while minimizing the risk of promoting a microbiome enriched with clinically significant resistance and virulence traits.

The differential expression results further reinforced the notion that protein intake modulates the expression of key microbial genes. More specifically, genes involved in methanogenesis and nitrogen fixation were upregulated in the high-protein group, reflecting the increased capacity for methane production and nitrogen utilization in response to elevated protein levels [32]. These results are in line with previous research [45,46] showing that dietary protein and energy contents influence the gene expressions of methanogens and other rumen microbes, highlighting the critical role of protein in regulating microbial metabolism [26]. In particular, the upregulation of methane-related genes emphasizes the need for a balanced approach to protein feeding in ruminants, as excessive protein intake may lead to higher methane emissions.

## 5. Conclusions

This study reveals that dietary protein levels significantly shape the gut archaeal community structure, diversity, and functional potential in sheep. Medium- and high-protein diets promoted greater archaeal alpha diversity and richness, with *Methanobrevibacter* and *Methanocorpusculum* species being dominant across groups. A moderate protein intake particularly supported a more diverse and balanced archaeal community, while high-protein diets enriched specific methanogenic species. Functional predictions indicated that dietary protein influenced archaeal pathways related to amino acid and carbohydrate metabolism, albeit with modest effect sizes. Antimicrobial resistance and virulence factor analyses further highlighted that high-protein diets favored the enrichment of clinically relevant resistance genes and virulence traits, suggesting potential implications for animal and public health. Overall, these findings emphasize the critical role of dietary protein in modulating gut archaeal ecology, resistome expansion, and functional adaptation, underscoring the importance of balanced protein formulations in livestock nutrition strategies. While in terms of microbial diversity and fermentation efficiency, the benefits of high-protein diets are evident, the increase in methane emissions presents a challenge for sustainable ruminant production systems. The current study presents limitations regarding dietary protein sources (plant versus animal origin) and their interactive action with other nutrients, such as energy. Therefore, further studies are warranted to investigate how dietary protein sources, and their interaction with other nutrients, can influence gut microbiota–host interactions, particularly in sheep.

## Figures and Tables

**Table 1:** The experimental dietary treatment formulation and its chemical composition (dry basis).

Variables	Treatments ^1^		
	H_l	H_m	H_h
Corn silage (%)	40.00	35.00	34.00
Wheat straw (%)	13.00	10.00	10.00
Bean powder (%)	2.00	10.00	11.00
Corn grain (%)	28.20	26.00	19.05
Soybean meal (%)	5.40	8.65	18.60
Corn starch (%)	8.65	7.60	4.70
Bean powder (%)	2.00	10.00	11.00
Premix (%) ^2^	1.00	1.00	1.00
Calcium carbonate (%)	0.55	0.60	0.65
Calcium hydrogen phosphate (%)	0.55	0.50	0.35
Salt (%)	0.30	0.30	0.30
Baking soda (%)	0.35	0.35	0.35
Forage to concentrate ratio	55:45	55:45	55:45
Chemical composition			
Dry matter (%)	89.06	88.92	88.75
ME (MJ/kg)	9.45	9.47	9.47
Crude protein (%)	8.58	10.34	13.93
NDF (%)	32.17	32.01	32.52
ADF (%)	17.24	17.71	18.44
EE (%)	2.39	2.32	2.43
Ash (%)	9.06	9.27	9.62
Calcium (%)	0.69	0.71	0.71
Phosphorus (%)	0.38	0.39	0.39

^1^ Treatments containing 8.58% (H_1), 10.34% (H_m), or 13.93% (H_h) protein levels. ^2^ One kg of premix contained the following: Ca: 151 g; Mg: 55 g; P: 85 g; I: 38 mg; Co: 9 mg; Cu: 65 mg; Fe: 5100 mg; Mn: 950 mg; Se: 6 mg; Zn: 890 mg; vita min A: 250,000 IU; vitamin D3: 35,000 IU; and vitamin E: 1550 IU.

**Table 2:** The values related to the observed species, Shannon, and Chao 1 index.

Group	Observed Species	Shannon	Chao1	Simpson	Goods Coverage
H_l	298 ± 25.53 ^b^	4.30 ± 0.59	317.18 ± 20.22 ^b^	0.89 ± 0.04	0.99 ± 0.00
H_m	394.67 ± 8.5 ^a^	4.79 ± 0.26	417.82 ± 4.39 ^a^	0.91 ± 0.02	0.99 ± 0.00
H_h	389.33 ± 39.07 ^a^	4.28 ± 0.67	425.91 ± 23.72 ^a^	0.89 ± 0.03	0.99 ± 0.00

Fecal samples of the lactating ewe groups fed low (H_l), medium (H_m), and high (H_h) dietary protein levels. Values with distinct superscripts (^a,b^) within a column indicate significant (*p* < 0.05) differences between groups, whereas those with the same superscript indicate non-significant differences (*p* > 0.05).

## Data Availability

The datasets generated and/or analyzed during the current study are available in the NCBI repository. The accessions for 16S rDNA included SAMN25981821, SAMN25981822, SAMN25981823, SAMN25981824, SAMN25981825, SAMN25981826, SAMN25981827, SAMN25981828, SAMN25981829, SAMN25981830, SAMN25981831, SAMN25981832, SAMN25981833, SAMN25981834, SAMN25981835, SAMN25981836, SAMN25981837, and SAMN25981838. The accessions for the metagenomic analysis included SAMN25981067, SAMN25981068, SAMN25981069, SAMN25981070, SAMN25981071, SAMN25981072, SAMN25981073, SAMN25981074, and SAMN25981075. The corresponding author can also provide data upon request.

## Supplementary Materials

The following supporting information can be downloaded at https://www.mdpi.com/article/10.3390/microorganisms13071560/s1, Table S1: Sequencing data validation.

## Abbreviations

The following abbreviations are used in this manuscript:

AMR	Antimicrobial resistance
CAZyme	Carbohydrate-active enzymes
CARD	Comparative antibiotic-resistant database
PCR	Polymerase chain reaction
VFDB	Virulence factor database
KEGG	Kyoto Encyclopedia of Genes and Genomes
