# Supplementary material for: Dietary Protein-Induced Changes in Archaeal Compositional Dynamics, Methanogenic Pathways, and Antimicrobial Resistance Profiles in Lactating Sheep
Source: Microorganisms. 2025 Jul 2;13(7):1560. doi: 10.3390/microorganisms13071560 (PMC12298320; doi:10.3390/microorganisms13071560)
Supplement: Supplementary file 1 [file microorganisms-13-01560-s001.zip › microorganisms-3711694-supplementary.pdf]

Table S1. Sequencing data validation

Group	Raw Data		Valid Data		Valid Ratio(reads)	Q20%	Q30%	GC content%	hostgenome rate%
	Read	Base	Read	Base					
H_m	126,059,758.0	6.30G	124,082,704.0	6.21G	98.43	99.66	98.18	44.50	2.67%
H_h	137,858,348.0	6.41G	125,995,648.0	6.30G	98.28	99.68	98.28	44.50	2.55%
H_l	119,147,206.0	6.45G	126,764,880.0	6.34G	98.23	99.70	98.35	44.67	2.29%
